# “The Critical 18” of postoperative white blood cell count and C-reactive protein level predicts postoperative pancreatic fistula after distal pancreatectomy

**DOI:** 10.1186/s13037-026-00497-9

**Published:** 2026-07-07

**Authors:** Ughur Aghamaliyev, Athanasios Zamparas, Stefanie Jarmusch, Gwendolin Seidel, Felix O. Hofmann, Gulnar Jafarova, Yannick Meyer, Simon Sirtl, Hanno Niess, Javad Karimbayli, Jens Werner, Bernhard W. Renz

**Affiliations:** 1 Department of General, Visceral and Transplantation Surgery, LMU University Hospital, Munich, Germany; 2 German Cancer Consortium (DKTK), partner site Munich, Munich, Germany; 3 Department of Medicine II, Ludwig Maximilians University Hospital, Munich, Germany; 4 Division of Molecular Oncology, Centro di Riferimento Oncologico di Aviano (CRO), IRCCS, National Cancer Institute, Aviano, Italy

**Keywords:** Pancreatic fistula, White blood cells, WBC, C-reactive protein, CRP, Pancreas, Fistula

## Abstract

**Background:**

Distal pancreatectomy (DP) carries high risk for postoperative pancreatic fistula. This study evaluates white blood cell count (WBC), C-reactive protein (CRP) and interleukin-6 (IL-6) as serum-based predictors, offering potential alternatives to drain amylase for early pancreatic fistula diagnosis.

**Methods:**

This retrospective cohort study included adult patients who underwent elective distal pancreatectomy between 01/2014 and 12/2023. Inclusion criteria required the availability of WBC count and CRP levels during the first three postoperative days. Exclusions were prior pancreatic surgery, combined DP/enucleation, or missing data. Data collected included demographics, comorbidities, intraoperative details, and postoperative serum markers (WBC, CRP, IL-6) from postoperative day 1 to postoperative day 3.

**Results:**

This study included 210 patients who underwent distal pancreatectomy and met all inclusion criteria. Median WBC counts were significantly higher in patients with pancreatic fistula, especially on postoperative day 2 (18.5 × 10⁹/L vs. 16.0 × 10⁹/L, *p* < 0,01). CRP levels peaked higher in the pancreatic fistula group on postoperative day 3 (19.8 mg/dL vs. 15.3 mg/dL, *p* < 0.001). IL-6 levels showed no significant difference. Receiver Operating Characteristic (ROC) analysis identified WBC on postoperative day 2 (AUC = 0.62) and CRP on postoperative day 3 (AUC = 0.65) as relevant predictors, with optimal cut-off values of 17.9 × 10⁹/L and 17.2 mg/dL, respectively, which were rounded to 18. The combined elevation of both markers, defined as the “Critical 18” was associated with a markedly higher pancreatic fistula rate (59% vs. 18% in patients without elevation of either marker).

**Conclusions:**

In light of these findings, we propose the “The Critical 18” as a simple yet powerful tool for early risk stratification of pancreatic fistula following distal pancreatectomy. By using WBC > 18 × 10 × 9/L on postoperative day 2 and CRP > 18 mg/dl on postoperative day 3, clinicians can easily identify patients at escalating risk, offering a clear framework for guiding postoperative management and interventions.

**Clinical trial number:**

Not applicable.

**Supplementary Information:**

The online version contains supplementary material available at 10.1186/s13037-026-00497-9.

## Background

Distal pancreatectomy (DP) is currently the only curative treatment option for pancreatic ductal adenocarcinoma (PDAC) localized in the pancreatic body or tail [1]. Advances in diagnostic techniques have led to an increase in the detection of pancreatic neuroendocrine tumors (pNET) and intraductal papillary mucinous neoplasms (IPMNs) in the pancreatic tail, resulting in more frequent indications for distal pancreatectomy [2, 3]. While DP is technically less complex than pancreatoduodenectomy (PD), it is associated with a higher incidence of postoperative pancreatic fistula (POPF) [4].

Postoperative pancreatic fistula is the severest complication after pancreatic resections and can lead to post-pancreatectomy hemorrhage, sepsis, organ failure and thereby postoperative mortality [4, 5]. Several factors including body mass index (BMI), pancreatic duct diameter, pancreatic texture and neck thickness, estimated blood loss and operation time were identified as risk factors of postoperative pancreatic fistula [6]. Although performing minimal invasive distal pancreatectomy led to better quality of life, the incidence of pancreatic fistula was not significantly different in comparison to open surgery [7]. Also, the effect of somatostatin and its analogues on exocrine pancreatic secretions was studied [8]. Moreover, studies have investigated whether patch plastics with fibrin sealants might reduce pancreatic fistula in patients undergoing DP or if omental wrapping could reduce their incidence [9]. Other techniques that have been studied over the years to reduce the pancreatic fistula involve closing the pancreatic stump either hand sewn or by using a stapling device [10]. In particular, in a randomised, controlled multicentre DISPACT trial with 450 patients, stapler closure did not reduce the postoperative pancreatic fistula rate compared to hand-sewn closure in DP, as both techniques had similar pancreatic fistula rates [11]. Most recently, the multicenter randomized controlled PANDORINA trial, showed that patients who did not have a drain inserted intraoperatively experiencing a significantly reduced rate of pancreatic fistula after distal pancreatectomy. This approach is now suggested as a new standard following DP [12].

Since drain amylase measurement was the primary diagnostic tool for detecting pancreatic fistula, serum markers are now emerging as appealing alternatives for predicting pancreatic fistula [13, 14]. In the context of PD, several studies have demonstrated effectiveness of serum markers in predicting the occurrence of pancreatic fistula [15]. Specifically, postoperative serum lipase and amylase levels have been shown to correlate with the risk of developing pancreatic fistula after both PD and DP [16].

Additionally, the importance of inflammatory markers such as white blood cell count (WBC), C-reactive protein (CRP) and interleukin-6 (IL-6) in predicting complications after PD have been studied previously [17, 18]. Studies evaluating the role of CRP levels over the first five postoperative days as a predictor of clinically relevant complications after PD have yielded significant results, with many indicating that CRP is a strong predictor of pancreatic fistula [19, 20]. CRP is generally a valuable tool predicting postoperative complications in abdominal surgery such as rectal resections and gastrectomies, where it has been shown being useful in anastomotic leakage prediction [21, 22]. CRP has also been studied in cases following DP, demonstrating promising diagnostic performance. In particular, two retrospective studies showed that elevated CRP values (> 140 mg/L in one study and > 180 mg/L in the second one) on postoperative day 3 were predictive markers for pancreatic fistula [23, 24]. Although, WBC are routinely examined postoperatively, studies shown controversial results regarding the predictive value of WBC in pancreatic surgery [25]. In contrast, IL-6 has been demonstrated to predict the development of pancreatic fistula after PD [26]. However, there are not enough data on the utility of IL-6 in patients undergoing distal pancreatectomy.

Therefore, the present study aims to address these controversies and gaps by investigating the potential of WBC, CRP, and IL-6 as predictive markers for postoperative pancreatic fistula after distal pancreatectomy.

## Methods

### Patients and study design

This single-center retrospective cohort study included patients who underwent distal pancreatectomy at our institution between 2014/01/01 and 2023/12/31. The analysis included patients who were 18 years or older and had undergone elective distal pancreatectomy. Inclusion criteria required the availability of WBC count and CRP levels during the first three postoperative days. Exclusion criteria included patients who underwent emergency surgery, had a history of previous pancreatic surgery, underwent DP with concomitant enucleation, or had incomplete or missing data.

### Data collection

Preoperative variables collected included demographic information, BMI, ASA classification (American Society of Anesthesiologists), history of alcohol abuse, smoking status, presence of cardiovascular and pulmonary comorbidities, preexisting diabetes, and prior administration of neoadjuvant therapy.

Intraoperative variables encompassed the type of surgery performed, operative duration, estimated blood loss, intraoperative blood transfusion requirements, and whether splenectomy, multivisceral resection, or vascular resection were performed. Additionally, the method of resection, either with a stapler or scalpel, was recorded.

Postoperative outcomes included histopathological findings, length of hospital stay, and any postoperative complications, with a focus on clinically significant issues such as pancreatic fistula, and 30-day mortality rates.

### Measurement of postoperative biochemical parameters

Blood samples were collected to measure postoperative WBC counts (×10⁹/L), CRP levels (mg/dL), and IL-6 concentrations (pg/mL) in all patients undergoing DP. These biomarkers were assessed daily as part of routine postoperative care, from the postoperative day 1 to postoperative day 3. The normal reference ranges at our institution were 3.9–9.8 × 10⁹/L for white blood cell count, < 0.5 mg/dL for C-reactive protein, and < 6 pg/mL for interleukin-6. For the analysis, all available data from the collected samples were utilized.

### Standard operative protocol

For patients diagnosed with tumors within the body to tail of pancreas, a distal pancreatectomy was performed. The ISGPS consensus statement was used to determine the standard lymphadenectomy protocol. To ensure complete resection, vascular resections and reconstructions were performed in cases of malignancies infiltrating the portal vein, superior mesenteric vein, or celiac axis. The intraoperative use of somatostatin analogues was determined by operating surgeon.

Distal pancreatectomy was conducted using either an open or minimally invasive approach and using either a stapling device or a scalpel dependent on the thickness of pancreatic parenchyma. In cases in which scalpel transection was used, the pancreatic duct was identified and subsequently closed with monofilament absorbable sutures. Abdominal drains were inserted at the end of the procedure.

### Postoperative management

Basically, cefuroxime was administered as a prophylactic antibiotic just before skin incision at operation by the majority of the patients. The postoperative use of somatostatin analogues as well as the duration of the use was determined by operating surgeon. The levels of drain lipase and amylase were measured in accordance with clinic standards. Drains were removed once enzyme levels decreased to less than three times the upper limit of normal. By patients with persistent pancreatic fistula till discharge day, the patients were discharged with the abdominal drains and were subject to weekly ambulant control. Pancreatic fistula is defined using the latest classification provided by the ISGPS [13]. Specifically, biochemical leak was identified as an elevation in drain amylase levels without any clinical impact. Patients exhibiting prolonged drainage for over 3 weeks due to heightened amylase activity were categorized as pancreatic fistula B. The classification of postoperative pancreatic fistula B was also conferred when interventions such as percutaneous, endoscopic, or angiographic procedures became necessary. In cases in which a pancreatic fistula B resulted in organ failure or clinical instability to the extent that reoperation is imperative, the pancreatic fistula B is upgraded to grade C. Furthermore, postoperative management protocols, including nasogastric tube (NGT) removal on postoperative day 1, resumption of oral intake, management of peripancreatic drains, and pain control, were performed in accordance with institutional standards. However, return of oral feeding included routinely liquid diet at postoperative day 1or 2. Then, if well tolerated, a semi-solid diet was given at postoperative day 2–3. No prokinetic agent was given as prophylaxis.

### Statistical analysis

Categorical variables were compared using the chi-squared test and Fisher’s exact test, and the Mann-Whitney U-test and Student’s t-test were used to compare continuous variables as appropriate. Receiver operating characteristic curve analysis was conducted using pROC package to evaluate the diagnostic performance of WBC, CRP, and IL-6 in distinguishing between patients with and without pancreatic fistula across the three postoperative days [27]. The area under the ROC curve (AUC) was calculated for each biomarker on each postoperative day to determine their predictive accuracy. The optimal cut-off values were identified with Youden Index [28]. For the univariate logistic regression analysis, both cut-off values of WBC and CRP were rounded to 18 to enhance their clinical applicability. Logistic regression analysis was used to obtain odds ratios (ORs) and 95% confidence intervals (CIs) and to examine the association between perioperative clinicopathological factors. Main variables, especially those showing significant results in the univariate analysis (*P* < 0.05), were subjected to multivariate binary logistic regression analysis. Statistical analysis and graphical illustrations were conducted using R-Software (Version 4.2.2).

## Results

### Study population

The study population comprised 210 patients who underwent elective distal pancreatectomy between January 2014 and December 2023 at our institution, and met the met inclusion criteria. Among them 54 (25.7%) developed pancreatic fistula. Notably, pancreatic fistula occurred more frequently in younger patients, with a median age of 59 years compared to 68 years in patients without pancreatic fistula (*p* < 0.001). Conversely, patients in the pancreatic fistula B/C group had a significantly higher median BMI (26 vs. 24, *p* = 0.004). Table 1 presents a comparative analysis of patient characteristics between those who developed pancreatic fistula and those who did not.

**Table 1:** Comparison of patient characteristics between patients with and without pancreatic fistula after DP

Variables	Overall (*n* = 210)	No-POPF (*n* = 156, 74.3%)	POPF B/C (*n* = 54, 25.7%)	*p*-value
Age	65 (56–74)	68 (59–75)	59 (55–66)	*< 0.001*
Sex, male	120 (57%)	88 (56%)	47 (50%)	0.9
Body mass index (kg/m^2^)	24.8 (22–28)	24 (22–27)	26 (23–30)	*0.004*
ASA ≥ 3	156 (74%)	114 (73%)	42 (78%)	0.8
Alcohol abuse	31 (18%)	25 (20%)	6 (14%)	0.4
Smoking	36 (22%)	27 (23%)	9 (20%)	0.17
Preoperative DM	50 (24%)	36 (24%)	14 (26%)	0.7
Cardiovascular diesases	95 (46%)	73 (47%)	22 (43%)	0.91
Pulmonary diseases	30 (15%)	23 (15%)	7 (13%)	0.13
NAT	59 (28%)	46 (30%)	13 (24%)	0.4
Tumor location				0.3
body	84 (40%)	65 (42%)	18 (35%)	
tail	126 (60%)	91 (58%)	36 (65%)	
Diagnosis				0.62
PDAC	82 (39%)	62 (40%)	20 (37%)	
pNET	40 (19%)	29 (19%)	11 (20%)	
Cystic lesions	29 (14%)	22 (14%)	7 (13%)	
CP	16 (7.6%)	10 (6%)	6 (11%)	
Others	43 (20%)	33 (21%)	10 (18%)	
Surgical approach				0.35
ODP	174 (82%)	129 (83%)	45 (83%)	
MIDP	31 (15%)	25 (16%)	6 (11%)	
Conversion	5 (2.4%)	2 (1.3%)	3 (6%)	
Pancreatic transection methods				0.6
hand sewn	84 (41%)	60 (40%)	24 (44%)	
stapler	119 (59%)	89 (60%)	30 (56%)	
Splenectomy	192 (91%)	143 (92%)	49 (91%)	0.4
Vascular resection	22 (11%)	18 (12%)	4 (7.0%)	0.4
Multivisceral resection	80 (38%)	62 (40%)	18 (33%)	0.6
Operation time	216 (167–273)	209 (168–271)	234 (161–296)	0.09
Estimated blood loss	500 (300–900)	500 (300–850)	550 (300–1000)	0.9
Intraoperative blood transfusion	22 (10%)	16 (10%)	6 (11%)	0.2
30-day mortality	1 (0.5%)	0 (0%)	1 (1.9%)	0.09

POPF: postoperative pancreatic fistula; DP: distal pancreatectomy; ASA: American Society of Anesthesiologists; DM: diabetes mellitus; NAT: neoadjuvant therapy; PDAC: pancreatic ductal adenocarcinoma; pNET: pancreatic neuroendocrine tumor; CP: chronic pancreatitis; ODP: open distal pancreatectomy; MIDP: minimal invasive distal pancreatectomy

### Postoperative biochemical differences between patients with and without pancreatic fistula

**Fig. 1:**
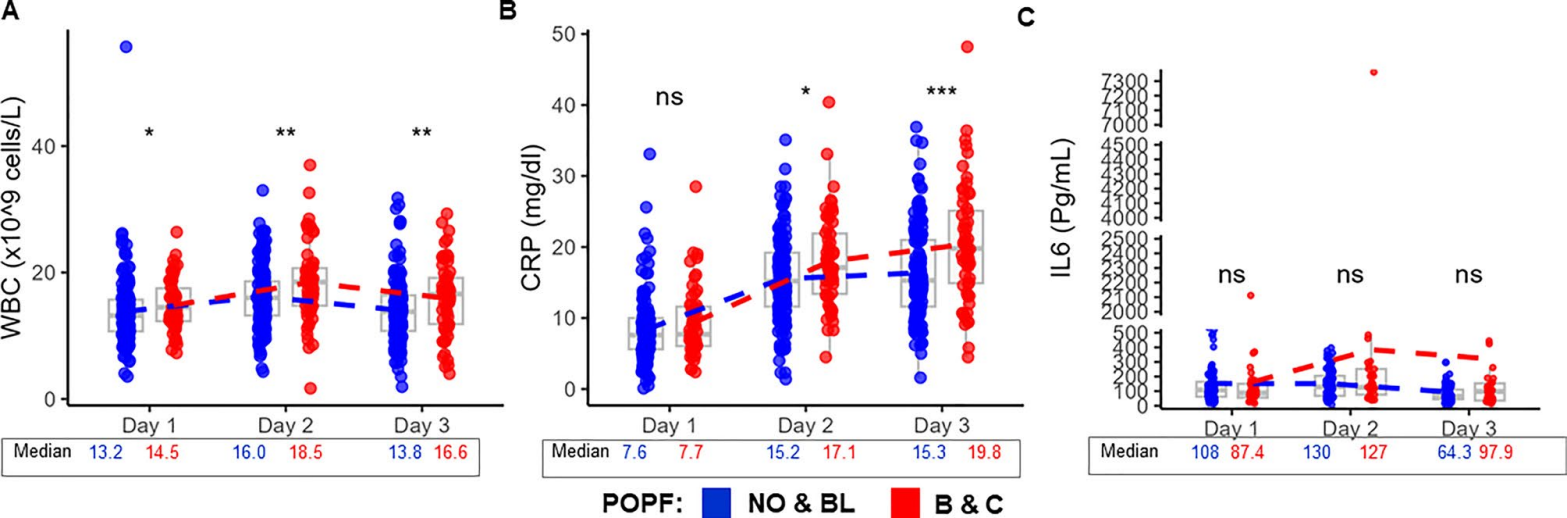
Postoperative biochemical differences between patients with and without pancreatic fistula. WBC: white blood cells; CRP: C-reactive protein; IL6: interleukin 6; POPF: postoperative pancreatic fistula; BL: biochemical leak

As shown in Fig. 1A, the median WBC count was significantly elevated in patients with pancreatic fistula compared to those without, throughout all three postoperative days. The highest WBC median in both groups occurred on postoperative day 2, followed by a gradual decline on postoperative day 3. Similarly, CRP levels exhibited a significant difference between the groups on postoperative day 2 and 3, but not on postoperative day 1, as shown in Fig. 1B. In both the no-POPF/BL group and the pancreatic fistula group, the highest median CRP levels were observed on postoperative day 3, reaching 15.3 mg/dL and 19.8 mg/dL, respectively. Interestingly, we observed no significant difference in IL-6 levels between both groups (Fig. 1C).

### Postoperative biochemical predictors for pancreatic fistula

**Fig. 2:**
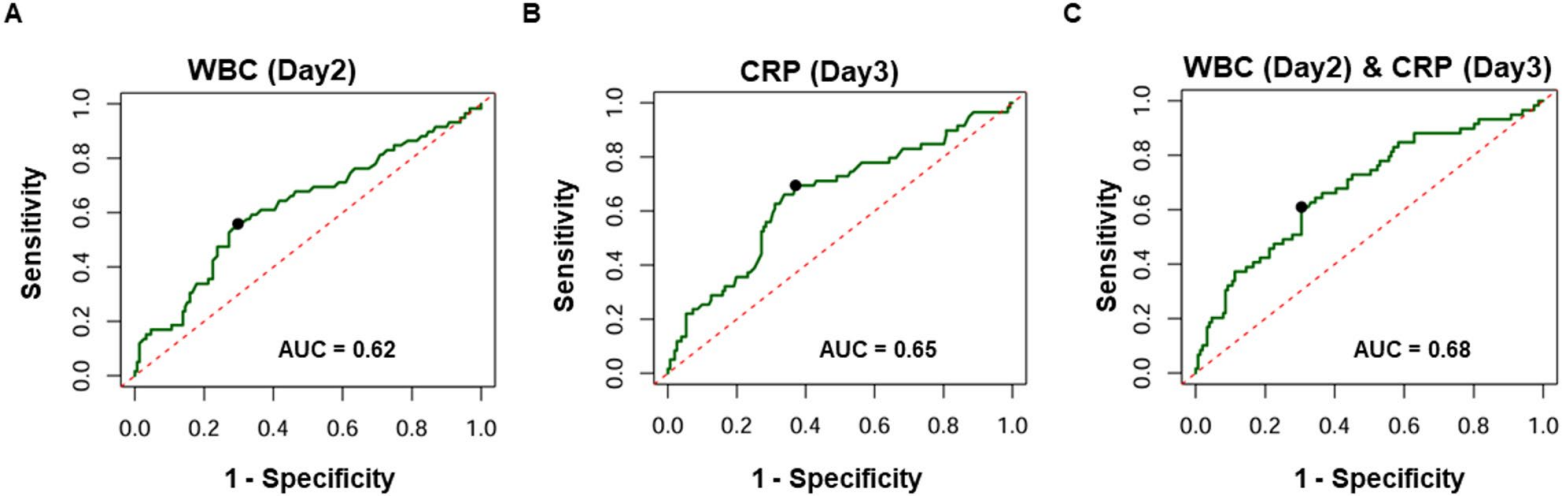
Receiver operative curves (ROC) for WBC on postoperative day 2 (**A**) and CRP on postoperative day 3 (**B**) and the combination of WBC on postoperative day 2 and CRP on postoperative day 3 (**C**) as predictors of pancreatic fistula. WBC: white blood cells; CRP: C-reactive protein

ROC analysis revealed that WBC, CRP, and IL-6 had varying degrees of diagnostic accuracy in predicting pancreatic fistula during the postoperative period (Fig. 2). Among the postoperative days, WBC showed the highest diagnostic performance on postoperative day 2 (Fig. 2A), with the largest area under the curve (AUC = 0.62) at an optimal cut-off value of 18.0, yielding a sensitivity of 56% and a specificity of 70% (Supplementary material Table S1). CRP showed its greatest predictive accuracy on postoperative day 3, achieving an AUC of 0.65 with an optimal cut-off of 17.25 mg/dL, providing a sensitivity of 69% and a specificity of 63% (Fig. 2B). The combination of WBC on postoperative day 2 and CRP on postoperative day 3 further improved diagnostic performance, resulting in an AUC of 0.68 (Fig. 2C). IL-6 had the weakest diagnostic performance in the ROC analysis, with its highest AUC on postoperative day 3 at 0.57, showing a sensitivity of 51% and a specificity of 69% (Supplementary material Table S1).

### Predictive value of WBC and CRP for pancreatic fistula

**Fig. 3:**
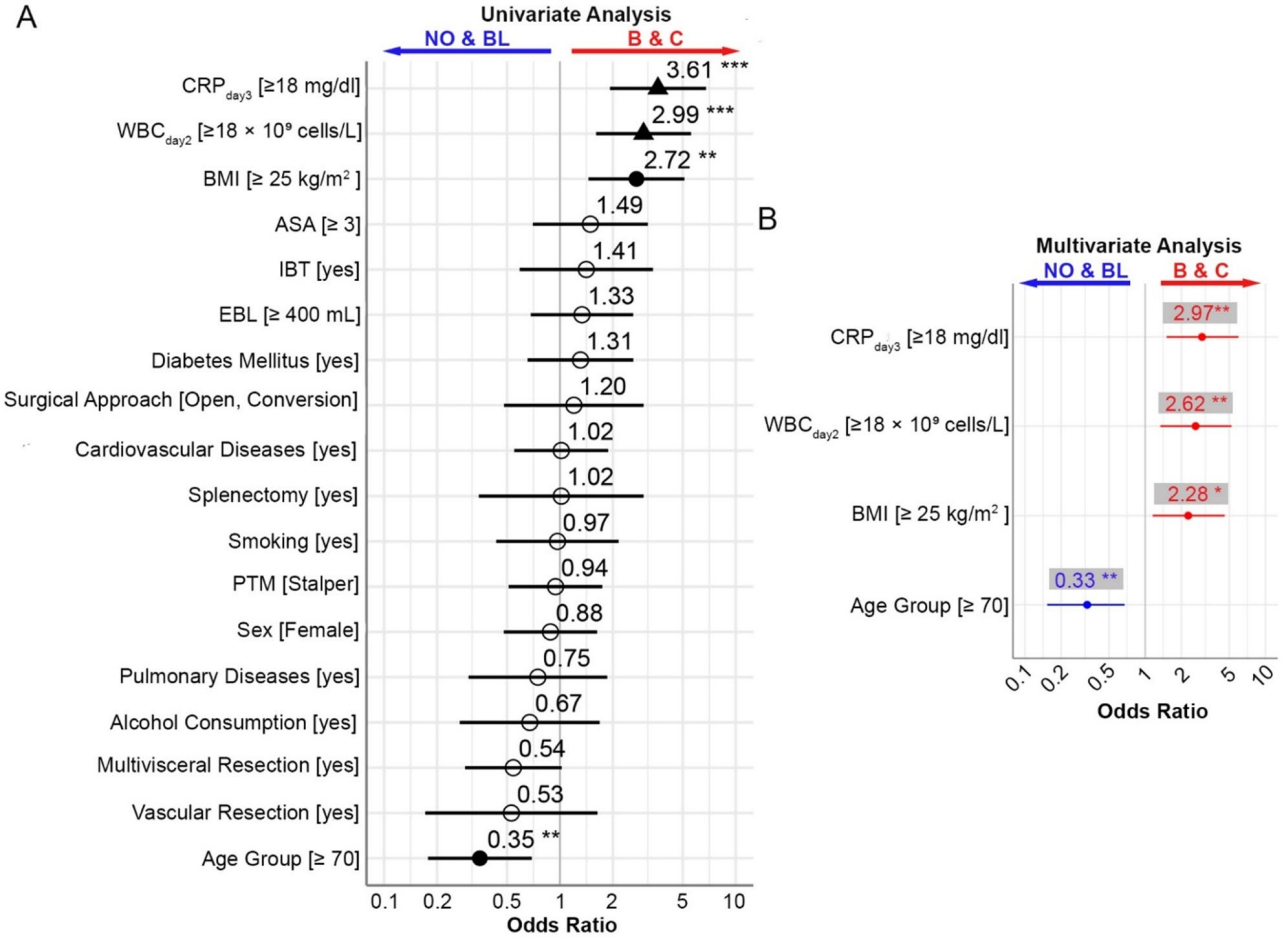
Univariate and multivariate analysis of pancreatic fistula after distal pancreatectomy. CRP: C-reactive protein; WBC: white blood cells; BMI: body mass index; ASA: American Society of Anesthesiologists; IBT: intraoperative blood transfusion; EBL: estimated blood loss; PTM: pancreatic transection method; BL: biochemical leak; B & C: postoperative pancreatic fistula B/C

The univariate logistic regression analysis revealed that a BMI over 25, age under 70, WBC > 18 on postoperative day 2, and CRP > 18 on postoperative day 3 were significantly associated with the development of pancreatic fistula (Fig. 3A). In the multivariate analysis, a BMI > 25 emerged as an independent risk factor for pancreatic fistula, while WBC on postoperative day 2 and CRP on postoperative day 3 were identified as independent predictive factors (Fig. 3B). Moreover, age > 70 was emerged as a significant protective factor for pancreatic fistula B/C.

### Incidence of pancreatic fistula stratified by “The Critical 18” of WBC and CRP

**Fig. 4:**
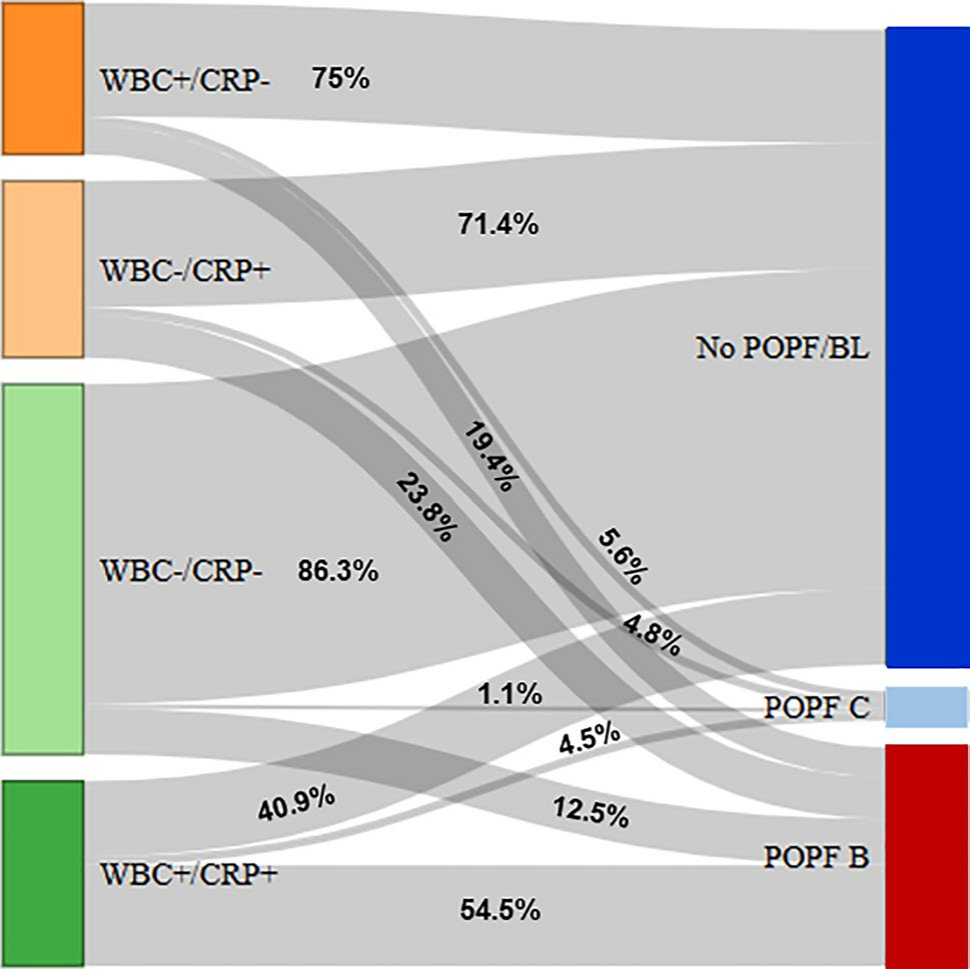
Incidence of pancreatic fistula in patients with and without “The Critical 18”. POPF: postoperative pancreatic fistula: BL: biochemical leak; WBC: white blood cells; CRP: C-reactive protein

Given that both WBC > 18 on postoperative day 2 and CRP > 18 mg/dL on postoperative day 3 were independent predictors of pancreatic fistula, their combination was defined as the “critical 18” criterion to facilitate risk stratification. When both parameters were considered, defined as “the critical 18”, the rate of pancreatic fistula increased to 59% (Fig. 4). In contrast, if neither of “the critical 18” thresholds were met, the pancreatic fistula rate decreased to 18% (Fig. 4).

## Discussion

In comparison to PD, DP is associated with a higher incidence of pancreatic fistula [29]. Numerous strategies have been explored to reduce the occurrence of pancreatic fistula following DP [4]. The most recent PANDORINA trial revealed that omitting intraoperative drain placement could safely reduce the incidence of pancreatic fistula after DP [12]. Traditionally, drain fluid analysis for lipase and amylase has been a key method for identifying patients at high risk of developing pancreatic fistula [13]. However, in cases where no intraoperative drain is inserted, postoperative serum markers may serve as promising alternatives for identifying these high-risk patients. In the present study, we demonstrated that “The critical 18” of WBC and CRP, defined WBC > 18 × 10⁹/L on postoperative day 2 and CRP > 18 mg /dl on postoperative day 3, can help to identify in early postoperative period patients at high risk for pancreatic fistula after DP.

WBC consist of several distinct subpopulations, with neutrophils being the most abundant [30]. Neutrophils are rapidly recruited to sites of acute inflammation, typically accumulating within hours to play a critical role in the initial immune response [30]. The predictive value of WBC count following DP remains controversial. On one hand, a WBC count greater than 16 × 10⁹/L on postoperative day 3 has been associated with major morbidity after DP in a cohort with 158 patients [31]. On the other hand, leukocytosis over 20 × 10⁹/L on postoperative day 1 has not shown a significant correlation with pancreatic fistula [25]. In contrast to previous studies, our cohort of 210 patients demonstrated that WBC counts were significantly higher in those who developed pancreatic fistula across the first three postoperative days. Thus, an elevated WBC count on postoperative day 2 may act as an early warning sign, suggesting the need for additional diagnostic steps, such as ultrasound, and early interventions, including initiating octreotide therapy, as soon as postoperative day 2.

CRP is an acute-phase protein primarily produced by hepatocytes [32]. In response to inflammation or tissue damage, CRP levels in the plasma rise within hours, peaking approximately after 48 h [33]. Connor’s widely accepted theory on postpancreatectomy pancreatitis incorporates CRP levels on postoperative day 2 exceeding 18 mg/dl as a key factor in assessing the severity of complications following pancreatic resections [34]. Furthermore, an increase in CRP by at least to 2.5 mg/dl on postoperative day 3 has been demonstrated as an independent predictor of pancreatic fistula after DP [24]. However, some other studies have also concluded that elevated CRP levels on postoperative day 3 might serve as a significant predictor of pancreatic fistula following DP [15]. E.g., in a cohort of 97 patients, CRP levels exceeding 14 mg/dl were identified as independent predictors of pancreatic fistula [23]. Similarly, a retrospective single-center study found that a higher threshold of 15.8 mg/dL for CRP on postoperative day 3 was an independent and significant predictor of postoperative intra-abdominal abscesses after DP [35].

In our study, CRP levels on postoperative day 3 showed the highest predictive performance among all three postoperative days. Patients with CRP levels ≥ 18 mg/dL on postoperative day 3 had a 2.4-fold higher likelihood of developing pancreatic fistula. Given that both WBC ≥ 18 × 10⁹/L on postoperative day 2 and CRP ≥ 18 mg/dl were significant independent predictors of pancreatic fistula in multivariate analysis, we propose the integration of these two valuable postoperative parameters into clinical practice as “The Critical 18.” In cases where both criteria are met, the risk of pancreatic fistula increases to 60%.

Another notable aspect of this study is the investigation of IL-6 in the large cohort of patients undergoing DP. IL-6 is a key cytokine in the innate immune response to inflammation and has demonstrated utility in predicting 28-day mortality in critically ill patients [36]. For this reason, IL-6 is frequently used in routine clinical practice. However, the evidence regarding the predictive value of IL-6 for pancreatic surgery-specific complications remains limited. According to our literature review, only two relevant studies have examined the role of IL-6 in the context of pancreatic surgery. A Dutch study involving 38 patients reported a correlation between IL-6 and pancreatic fistula after PD [37]. Another study, with a small cohort of 43 PDs and 27 DPs, found that IL-6 on postoperative day 3 was a significant predictor of pancreatic fistula [26].

In our cohort, 169 patients had serum IL-6 levels measured on postoperative day 1, making this the largest study of its kind. However, we did not observe a significant predictive value for IL-6 in predicting pancreatic fistula after DP. Nevertheless, further research is necessary, particularly regarding the predictive value of IL-6 following PD, total pancreatectomy, or enucleation.

While this study offers a valuable and unique approach by analyzing WBC, CRP, and IL-6 over three postoperative days, it does have some limitations. The retrospective design may introduce some observational challenges, and IL6 levels were not always available on every day for all patients, which might affect data consistency.

## Conclusions

In conclusion, our study emphasizes the importance of “The Critical 18” as a reliable tool for early identification of high-risk patients for pancreatic fistula following DP. The combined criteria of WBC > 18 × 10⁹/L on postoperative day 2 and CRP > 18 mg/dL on postoperative day 3 provide a pragmatic and effective framework for timely clinical decision-making. By enabling early recognition and intervention, this approach holds significant potential to mitigate fistula-related complications, ultimately reducing morbidity and mortality. While the role of IL-6 remains uncertain in this context, our findings support further exploration into the utility of these biomarkers across various types of pancreatic surgery.

## Supplementary Information

Below is the link to the electronic supplementary material.

Supplementary Material 1

## Data Availability

The datasets analyzed during the current study are not publicly available due to institutional data protection regulations but are available from the corresponding author on reasonable request.

## Abbreviations

ASA: American Society of Anesthesiologists
BMI: Body mass index
CI: Confidence interval
CRP: C-reactive protein level
CT: Computed tomography
DP: Distal pancreatectomy
IL-6: Interleukin-6
IBT: Intraoperative blood transfusion
ISGPS: International Study Group of Pancreatic Surgery
MIDP: Minimally invasive distal pancreatectomy
NAT: Neoadjuvant therapy
NGT: Nasogastric tube
OR: Odds ratio
PD: Pancreatoduodenectomy
PDAC: Pancreatic ductal adenocarcinoma
pNET: Pancreatic neuroendocrine tumor
POPF: Postoperative pancreatic fistula
POD: Postoperative day
PTM: Pancreatic transection method
ROC: Receiver operating characteristic
WBC: White blood cell count
