# Supplementary material for: “The Critical 18” of postoperative white blood cell count and C-reactive protein level predicts postoperative pancreatic fistula after distal pancreatectomy
Source: Patient Saf Surg. 2026 Jul 7;20:26. doi: 10.1186/s13037-026-00497-9 (PMC13355360; doi:10.1186/s13037-026-00497-9)
Supplement: Supplementary file 1 — Supplementary Material 1 [file 13037_2026_497_MOESM1_ESM.docx]

| **Marker** | **n** | **Estimate** | **Std_Error** | **z_value** | **p_value** | **AUC** | **CI_Lower** | **CI_Upper** | **threshold** | **sensitivity** | **specificity** |
| --- | --- | --- | --- | --- | --- | --- | --- | --- | --- | --- | --- |
| **WBC POD1** | 210 | 0,03413083 | 0,02933017 | 1,16367652 | 0,24455513 | 0,58900585 | 0,503735 | 0,67427669 | 14,45 | 50,877193 | 66,6666667 |
| **WBC POD2** | 210 | 0,07939062 | 0,02972712 | 2,67064599 | 0,00757054 | 0,62414412 | 0,53635463 | 0,71193362 | 17,9 | 55,9322034 | 70,1986755 |
| **WBC POD3** | 210 | 0,06413463 | 0,02853345 | 2,24769995 | 0,02459533 | 0,61768998 | 0,5275585 | 0,70782145 | 16,55 | 50,8474576 | 75,4966887 |
| **CRP POD1** | 210 | 0,04112247 | 0,03191686 | 1,28842493 | 0,19759808 | 0,55159542 | 0,46132792 | 0,64186292 | 11,35 | 27,2727273 | 85,4304636 |
| **CRP POD2** | 210 | 0,06416108 | 0,02542356 | 2,52368623 | 0,01161315 | 0,605335 | 0,5202234 | 0,6904466 | 15,05 | 69,4915254 | 48,9932886 |
| **CRP POD3** | 210 | 0,07760468 | 0,02219783 | 3,49604832 | 0,0004722 | 0,65321585 | 0,56828732 | 0,73814438 | 17,25 | 69,4915254 | 62,9139073 |
| **IL6 POD1** | 169 | 0,00020109 | 0,00072544 | 0,27720185 | 0,78162512 | 0,54896116 | 0,44843868 | 0,64948364 | 104,5 | 62,2222222 | 55,2845528 |
| **IL6 POD2** | 136 | 0,00219168 | 0,00144933 | 1,51220542 | 0,13048161 | 0,45849421 | 0,34545418 | 0,57153424 | 127,5 | 51,3513514 | 52,0408163 |
| **IL6 POD3** | 119 | 0,00195807 | 0,00141395 | 1,38482485 | 0,16610603 | 0,57040998 | 0,44459653 | 0,69622344 | 97,1 | 51,5151515 | 69,4117647 |

**Supplementary Material Table S1.** Diagnostic performance of postoperative inflammatory markers (WBC, CRP, IL-6) measured on postoperative days (POD) 1–3. Shown are n, regression results (estimate, standard error, z-value, p-value), AUC with 95% CI, and corresponding cutoff, sensitivity, and specificity.
